# Recent Advancements in Fabrication of Metal Matrix Composites: A Systematic Review

**DOI:** 10.3390/ma17184635

**Published:** 2024-09-21

**Authors:** Pallab Sarmah, Kapil Gupta

**Affiliations:** Department of Mechanical and Industrial Engineering Technology, University of Johannesburg (Doornfontein Campus), Johannesburg 2028, South Africa; psarmah@uj.ac.za

**Keywords:** MMCs, stir casting, powder metallurgy, additive manufacturing, FSP, machine learning

## Abstract

Metal matrix composites (MMCs) are essential materials in various industries due to superior properties, such as high strength-to-weight ratios, better corrosion resistance, improved wear resistance and adaptability, developed by continuous improvements in their fabrication methods. This helps to meet the growing demand for high-performance and sustainable products. The industries that stand to gain the most are automotive and aerospace, where MMCs are used for car parts, airplane frames, and jet engines that need to be strong and lightweight. Furthermore, MMCs are being extensively used in the biomedical industry for implants and medical equipment because of their suitable mechanical integrity and corrosion resistance. Applications in heavy construction, defense, and even space exploration are noteworthy. The advancements in fabrication of MMCs have revolutionized the composite industry with their improved mechanical, tribological, and metallurgical properties. This review article offers an introduction and thorough examination of the most recent advancements (mostly within the last five years) in fabrication methods of MMCs. The novelty and modernization in the traditional processes and advanced processes are covered, along with discussing the process parameters’ effects on the microstructure and properties of the composites. The review focuses on features and prospective applications of MMCs that have been greatly improved and extended due to such advancements. The most recent methods for developing MMCs, including friction stir processing (FSP), ultrasonic-assisted stir casting, and additive manufacturing, are discussed. Artificial intelligence and machine learning interventions for composite manufacturing are also included in this review. This article aims to assist researchers and scholars and encourage them to conduct future research and pursue innovations to establish the field further.

## 1. Introduction

### 1.1. Overview of Metal Matrix Composites

**Figure 1:**
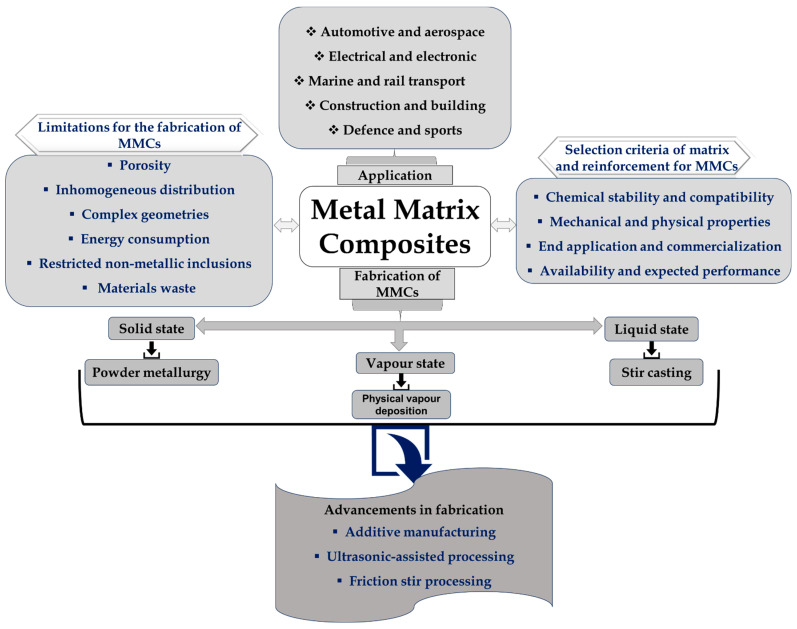
Overview of fabrication of metal matrix composites.

Metal matrix composites (MMCs) are a class of materials comprising a metal or alloy matrix reinforced with particles, whiskers, fibers, or hollow micro balloons. The reinforcement materials, which can be metallic, ceramic, or polymeric, are added to boost mechanical properties, thermal stability, and wear resistance of the base metal. When compared to other composites, MMCs have a distinct set of properties. Among all MMCs, some composites are perfect for applications in a diversity of industries, including automotive, aerospace, and transportation, since they have a high strength-to-weight ratio, a low thermal expansion coefficient, and great wear and abrasion resistance [1]. MMCs can offer better property combinations than traditional composites, particularly when hybrid MMCs with various reinforcements are used. MMCs are energy-saving materials that are widely employed because of their acknowledged capacity to deliver required qualities such as tensile strength, stiffness, hardness, and fatigue and wear resistance [2]. Modern materials with high-temperature resistance, particular stiffness, and specific strength have been developed as a result of the switch from monolithic metals to advanced materials like MMCs [3]. For this reason, MMCs are essential engineering materials for sectors like the automotive, aerospace, defense, and marine industries. Furthermore, MMCs’ self-healing qualities help to extend the material’s life and dependability, particularly in aeronautical applications [4]. The synthesis and production of MMCs have been achieved using a variety of manufacturing techniques. During the fabrication of MMCs, exact control over the distribution of reinforcement components inside the metal matrix is essential for several reasons. The distribution of reinforcing components has a major impact on the mechanical properties of MMCs, including strength, stiffness, and toughness. These qualities are improved by uniform distribution, but a non-uniform distribution may result in high-stress concentrations or isolated weaknesses that lower the composite’s overall performance. Precise management of reinforcement distribution is necessary for several manufacturing processes, including casting, additive manufacturing, and others, to guarantee the end product’s quality and uniformity. For instance, selective laser melting (SLM) produces improved mechanical characteristics by enabling exact control over fiber distribution. A homogenous distribution of reinforcements is necessary to achieve the desired qualities in MMCs. For applications needing consistent performance, non-uniform distribution might result in differences in material qualities across the composite’s various components. The reinforcing elements must interact with the metal matrix. The strength and durability of the composite are influenced by the optimal interface bonding, which is achieved by precise control over distribution. Many techniques were used to enhance the outcomes, but the main goal was to provide a practical, affordable, and effective processing method where the characteristics would be changed. Choosing the right fabrication method is essential because it affects the microstructural characteristics, including the distribution, orientation, and interfacial attachment among reinforcement and matrix [5]. Based on research papers supplied, several viable solutions can be taken into consideration to advance the efficiency of MMC manufacturing processes. An overview of MMCs with the applications and fabrication processes is presented in Figure 1.

The benefits and drawbacks of these processes are limited by the specifications and features of the final product. Liquid state, solid state, and vapor state processes are three groups into which the basic techniques are divided. The current methods for MMC fabrication are limited in terms of cost and scalability due to problems with traditional routes such as liquid and powder metallurgy, where particles may not sufficiently wet the molten metal, settle or float because of density variations, and form unwanted intermetallic particles [6]. Furthermore, the creation of a strength-depleted zone along joint lines limits component size when combining MMCs via fusion welding. Although stir casting and powder metallurgy processes are widely employed in the production of MMCs, the need for sophisticated production techniques such as additive manufacturing (AM) is emphasized to get around these constraints and improve scalability [7]. This highlights the significance of resolving technical challenges for future applications of MMCs. One of the most important factors influencing MMCs’ performance during the intricate preparation process is their structure. The distribution, size, and type of reinforcement in the structure, along with the matrix material, have a major impact on the mechanical characteristics and overall performance of the MMCs. Performance is impacted by the structure in the following significant ways:

- Mechanical properties:
  *Strength and stiffness:* MMCs’ strength and stiffness are enhanced by the addition of reinforcements, such as fibers or ceramic particles. Since the reinforcements are usually stiffer and stronger than the matrix material, the load is transmitted from the matrix to them.
  *Toughness:* MMCs’ toughness is influenced by the type and distribution of reinforcement. By stopping the spread of cracks, reinforcements arranged uniformly can increase durability.
- Wear resistance:
  *Surface hardness*: The wear resistance of MMCs is enhanced by the hardness of the reinforcing elements, such as ceramic particles. Wear during operation can be decreased by a tougher surface created by a well-dispersed reinforcing structure.
  *Load distribution:* By minimizing localized stress concentrations and increasing wear resistance, a uniform distribution of reinforcements aids in a more even distribution of the load.
- Thermal properties:
  *Thermal conductivity:* MMCs’ thermal conductivity is influenced by their structural face, namely the kind and arrangement of reinforcements. For example, adding carbon fibers can improve MMCs’ heat conductivity.
  *Thermal expansion:* The reinforcing structure has an impact on the coefficient of thermal expansion (CTE) of MMCs. A well-designed structure can minimize thermal expansion mismatch between the matrix and reinforcements, improving dimensional stability.
- Fatigue performance:
  *Crack initiation and propagation:* MMCs’ fatigue performance is influenced by their structure, particularly the interface between the matrix and reinforcements. A strong interface can inhibit crack start and propagation, improving fatigue life.
  *Load transmission:* An essential component of fatigue performance is the reinforcements’ capacity to efficiently transmit load to the matrix. Effective load transmission is ensured by a well-bonded contact, which lowers the possibility of fatigue failure.
- Corrosion resistance:
  *Protective layers:* To increase corrosion resistance, protective layers or coatings on the reinforcements can be included in the construction of MMCs. Coatings, for instance, can stop galvanic corrosion between reinforcements and the matrix.
  *Barrier effect:* Corrosive agents may find it more difficult to permeate the matrix material when reinforcements are evenly distributed.
- Processing and manufacturability:
  *Flow properties:* During processing, the size and form of the reinforcements, in particular, have an impact on the flow properties of MMCs. A uniform distribution can be more easily achieved with smaller particles due to their improved flowability.
  *Defect formation:* Agglomeration and porosity are two examples of flaws that the structure may affect. By reducing these flaws, advanced manufacturing methods, including friction stir casting and ultrasonic-assisted stir casting, enhance the overall quality and functionality of MMCs.
- Interface properties:
  *Bonding strength:* The effectiveness of MMCs depends critically on the interaction between the matrix and reinforcements. Improved mechanical qualities and effective load transmission are guaranteed by a robust interface.
  *Chemical compatibility:* The qualities of the interface are influenced by the matrix and reinforcements’ chemical compatibility. Improved interfacial bonding can be facilitated by sophisticated processing methods, improving MMCs’ overall performance.

### 1.2. Review Methodology

The first step in the review process is to specify the goals and parameters of the review, which is centered on analyzing the most recent developments in fabrication of MMCs over the last five years, with a special focus on advancements in solid and liquid methods for the fabrication of MMCs. Additionally, the scope includes novel and advanced techniques used for the fabrication of MMCs. A list of precise keywords, including “Advancements in fabrication of MMCs” and “Novel techniques for fabrication of MMCs”, was formed to start the literature search. The search method across the most important academic databases, such as Scopus and Google Scholar, was conducted with these keywords. An initial broad search utilizing specific keywords was part of the search process, and it produced a significant number of research papers from a variety of disciplines. With the use of this technique, the results were restricted to research that dealt directly with the advancements in the fabrication of MMCs during the previous five years.

In order to synthesize the examined literature, the findings had to be analyzed and framed in connection to the contents of the article. This involved extending the scope from traditional methods to advanced techniques employed for the fabrication of MMCs. The methodology included a critical component for identifying the advantages and limitations of each fabrication method. To assist academics and industry professionals with good-quality MMC products, the analysis offered suggestions for this review.

The methodology placed a strong emphasis on the necessity of precisely establishing search parameters and avoiding the omission of important material to guarantee an inclusive and thorough search. The search strategy was created to be both broad and specialized enough to include a large number of applicable studies, while still producing relevant results. This strategy was essential for giving a full and accurate analysis of the most recent developments in the fabrication of MMCs in detail.

In summary, a mixed systematic approach to the search, evaluation, and synthesis of the literature is used in the methodology of this review article. The approach seeks to offer insightful analysis and further the body of knowledge in the sector by emphasizing current developments and the integration of advanced techniques for the fabrication of MMCs over the traditional methods.

The goals of this review can be summed up as follows:

❖ To gather and examine literature on recent implementations for the fabrication of MMCs over the previous five years, with a focus on improving the quality of composite products.
❖ To examine the outcomes and determine how the latest developments help to achieve good-quality MMCs with enhanced physical properties.
❖ To recognize and explore the effects of fabrication parameters on MMCs in the existing literature concerning their applications in various industries.
❖ To present a thorough synthesis of the results and provide an in-depth understanding of the major issues, developments, and trends in advancements for fabrication of MMCs.

## 2. Advances in Fabrication Methods of MMCs

### 2.1. Advances in Solid-State Processes

**Figure 2:**
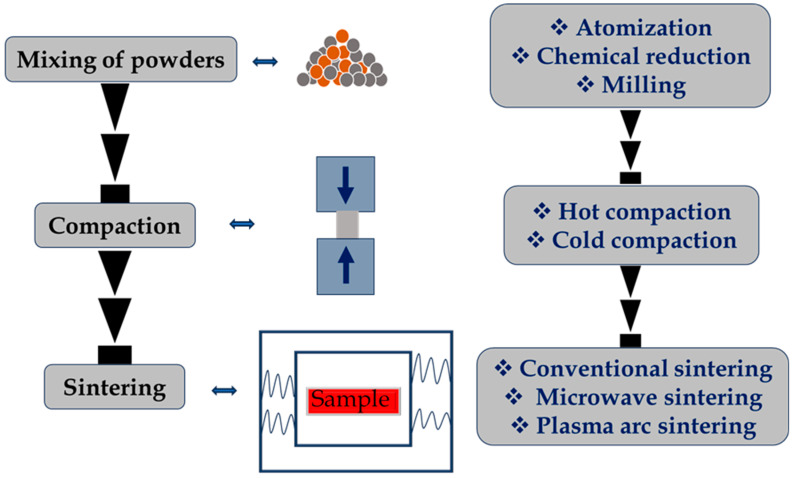
Illustration of solid-state fabrication of MMCs.

MMCs are used in many different sectors, as there is growing demand globally. The right reinforcing materials and production techniques may help MMCs have the appropriate qualities [8]. Based on solid phase processing, the powder metallurgy (PM) technique is the most frequently used. This typically contains discontinuous reinforcements since mixing and densification are simple [9]. To obtain the highest density, metal particles are mixed, statically cold-compressed, and then heated. The fully dense compact is then often subjected to a subsequent process like forging or extrusion. In PM, before cold pressing and sintering, reinforcing materials (particles or fibers) are combined with base matrix (pure metal or alloy) material powders. This happens in a dry setting, with the aid of dispersion agents, or under controlled conditions through mechanical alloying. Most of the time, moderate temperatures are used for PM operations, and there is little to no interaction between the components of the matrix and reinforcement [10]. The insertion of short fibers or whiskers may require the use of microscopic particles to increase the scattering and filling of the microstructure, although uniformly dispersing reinforcing phases within the matrix phase can provide composites with greater mechanical properties. PM has been a traditional method for fabricating MMCs. It involves blending metal powders with reinforcement materials, followed by compaction and sintering. Recent advancements in PM include the use of high-energy ball milling to enhance distribution of reinforcement particles and application of spark plasma sintering (SPS) for faster and more efficient consolidation. When it comes to producing MMCs, PM has great promise since it offers more control over the microstructure and characteristics of the material than traditional techniques do [11]. This results in better mechanical properties and a greater resistance to deformation. Figure 2 demonstrates the solid-state fabrication process for MMCs.

A practical and affordable way to develop MMCs with superior mechanical and tribological characteristics is through the PM process [12] Improved mechanical and tribological characteristics of the composites can result from a careful choice of process parameters, for example, compaction pressure, sintering time, and temperature. It is crucial to research mechanical and wear characteristics of particle-reinforced MMCs. Techniques like powder blending, surface modification, optimized sintering parameters, advanced consolidation methods, and the development of new strategies like double pressing-double sintering (DPDS), and hot pressing (HP) are examples of novel and advanced strategies used in the PM process for MMCs. These strategies and techniques seek to overcome difficulties that arise during the production of MMCs, including distributing powder uniformly, improving bonding across surfaces, avoiding particle agglomeration, managing porosity, and guaranteeing appropriate dispersion of reinforcement. Furthermore, the field of MMC fabrication advances with the usage of hybrid reinforcements like carbon nanotubes (CNTs) and graphene (Gr). PM and traditional sintering techniques can be used to create copper-graphite composites with good mechanical characteristics [13]. As the percentage of Gr rises, wear resistance improves, reaching an 88% improvement. At 1% Gr concentration, microhardness and compressive strength are at their peak, after which they start to decline. Using mechanical alloying, milling, and two different powder metallurgy methods, DPDS and HP, Sterioudi et al. [14] successfully produced an AA 2024 alloy composite reinforced with MWCNTs. The HP method produced the best mechanical properties, but clustering caused a dramatic decrease in properties above 2 wt.% MWCNT content. Comparing the AA2024/MWCNT nanocomposites to pure AA2024, both the DPDS and HP processes produced better mechanical characteristics; however, because of their greater density and finer grain, the HP approach produced better mechanical properties overall. The MWCNTs’ inhomogeneous dispersion and clustering were responsible for the sharp decline in mechanical characteristics that occurred when the MWCNT level exceeded 2 wt.%. Both methods can yield nanocomposites with good strength–ductility balance. The DPDS method produced the AA2024-2 wt.% MWCNT nanocomposite, which had a compressive strength of 350 MPa and 16% elongation, while the HP method produced the same nanocomposite, which had a compressive strength of 423 MPa and 19% elongation.

Haque et al. [15] examined the impact of aging and PM process parameters on the wear and hardness characteristics of Al 6063 MMCs reinforced with different volumes of nano Al_2_O_3_ particles. As the proportion of nano Al_2_O_3_ addition increases, the wear rate of the Al6063-nano Al_2_O_3_ MMCs decreases; the 8% nano Al_2_O_3_ composition exhibits the lowest wear rate. The ideal processing conditions are 550 MPa of compaction pressure and 60 min of compaction time to minimize wear rate and maximize microhardness. Up to a 6% addition of nano Al_2_O_3_, the microhardness of the MMCs rises; however, at an 8% addition, it decreases. Ni-base MMCs were created using PM hot isostatic pressing and are intended for use in spacecraft mechanical seals [16]. It was discovered that the volume proportion of ceramic reinforcement (SiC or TiB_2_) in the IN625 matrix was important; greater fractions resulted in improved tribological characteristics and enhanced hardness. The optimal blend of tribological characteristics, machinability, and hardness was found in IN625-10 %SiC, which was employed to fabricate two mechanical seal demonstrators with success. Zamani et al. [17] studied tribo-mechanical features of self-lubricating hybrid MMCs based on Al and reinforced with Gr and Al_2_O_3_. The hybrid MMCs, particularly the ones with 10% and 3% Al_2_O_3_ and Gr, have much better tribological and mechanical properties than the base materials. An Al-Gr composite’s tribological characteristics, such as wear rate and coefficient of friction (COF), as well as its mechanical characteristics, such as hardness, tensile strength, and flexural strength, were greatly enhanced by the addition of Al_2_O_3_. To better understand the mechanical and physical characteristics of Al-based MMCs created through PM, Nayak et al. [18] examined four distinct hybrid composite samples that were created by combining aluminum powder with various ratios of Al_2_O_3_ and SiC. Materials with the combination of (Al/2%SiC/3%Al_2_O_3_) and (Al/7%SiC/3%Al_2_O_3_) exhibited higher density and mechanical characteristics after one hour of optimum sintering at 700 °C. Reminder additions in excess (>3% Al_2_O_3_, >8% SiC) resulted in decreased density, hardness, and compressive strength as well as poor sinterability.

Al-based MMCs were fabricated with TiO_2_ with the use of PM methods and investigated to characterize the material’s mechanical and microstructure features and to produce a strong, lightweight, and affordable material [19]. As the TiO_2_ concentration rose from 0 to 12 wt.%, the Al-TiO_2_ composites’ compressive strength increased from 67.90 MPa to 77.53 MPa, and Brinell hardness increased from 19.51 BHN to 26.08 BHN. The mechanical characteristics of Al-SiC functionally graded materials (FGMs) are used for braking applications [20]. Compared to five-layered FGM specimens, four-layered specimens demonstrated superior mechanical characteristics and a more uniform microstructure. Over-reinforcement of SiC in the Al matrix can cause brittleness and a decrease in toughness of the material. Tosun and Kurt [21] conducted preparation and analysis of Al-Mg MMCs augmented with a PM process, micro-sized SiC or Al_2_O_3_ particles. The 15% SiC/Al-Mg composite generated at 300 MPa and 400 °C for 90 min had a maximum porosity ratio of 17%, while 15% SiC/Al-Mg composite produced at 600 MPa and 500 °C for 90 min had the lowest porosity ratio of 5.4%. Raising the sintering temperature from 300 °C to 400 °C resulted in a decrease in the microhardness of the composite in 15% SiC and Al_2_O_3_/Al-Mg composites. Reaching 500 °C during the sintering process increased microhardness for 15% SiC and Al_2_O_3_/Al-Mg composites. The microhardness of 30% SiC/Al-Mg composites dropped with increasing sintering temperature. Agglomeration inside the structure and increased Al_4_C_3_ phase production are caused by rising sintering temperatures. The quantity of microparticles is decreased by the reinforcement’s clustering. Consequently, the microhardness and strength decreased. As SiC is harder than Al_2_O_3_, Al-Mg composites are harder than SiC/Al-Mg composites.

In another work, Parveen et al. [22] utilized a PM method to manufacture Al-based hybrid composites reinforced with Si_3_N_4_ and ZrO_2_. The Al-Si_3_N_4_/ZrO_2_ composite showed improved density and hardness, reduced porosity, and more uniform particle distribution as a result of increasing the milling duration and wt.% of the nano-ZrO_2_ reinforcement. Copper matrix composites’ hardness, wear resistance, and corrosion resistance were all increased by the inclusion of WC and SiC reinforcements [23]. The addition of alloying materials to Cu-base matrix enhanced electrical conduction of the composites. With the addition of Cu, composites’ resistance to corrosion is enhanced. HP was used to effectively create AZ91 matrix composites containing 10, 20, and 30% TiB_2_ [24]. The composites outperformed the unreinforced AZ91 alloy in terms of hardness, wear resistance, and mechanical characteristics. The TiB_2_ particles were distributed uniformly, although at 30% there was some partial agglomeration. Due to micropores, the relative density of the composites decreased as the TiB_2_ concentration increased, while wear resistance and hardness considerably increased. While the failure strain of the composites was higher than that of the AZ91 alloy, the compressive yield strength and ultimate compressive strength increased with TiB_2_ content up to 20%. When a large volume of 60% glassy particles based on Fe was used as reinforcement in an Al matrix, the Al matrix was significantly hardened while retaining its excellent strength and flexibility [25]. The composites demonstrated effective densification without unfavorable effects at the matrix–reinforcement interfaces by achieving high relative densities of 97–98% with relatively few apertures. According to a microstructural study, greater SiC levels caused agglomeration, whereas the Al+4wt%SiC composite exhibited the most uniform distribution of reinforcement [26]. The hybrid composite Al+4wt.%SiC+2wt.%B_4_C exhibited the best interfacial bonding between matrix and reinforcement, as well as a homogeneous structure. Under both 5N and 10N loads, the Al+4%SiC+2%B_4_C hybrid composite had the maximum compressive strength, while the Al+5%SiC+2%B_4_C hybrid composite had the lowest wear rate. Mg/B_4_C composites containing 10, 20, and 30% B_4_C were effectively created using HP and PM methods [27]. The composites’ compressive yield strength, hardness, and wear resistance all significantly increased with the inclusion of B_4_C particles; Mg/30 wt.% B_4_C composite had the finest wear resistance.

While examining corrosion behavior of Al/SiC composites made using PM methods, from 10 to 20% SiC content resulted in higher corrosion rates and also improved resistance to pitting corrosion [28]. Once related to uniform corrosion in chloride conditions, the pitting corrosion resistance of Al/SiC composites improved with increasing SiC (10–20%) content. Al-based MMCs reinforced with SiC and Al_2_O_3_ particles were synthesized and characterized using the PM process [29]. Aluminum’s compressive strength was increased by adding SiC and Al_2_O_3_ reinforcements; however, adding more reinforcements than the recommended 7% SiC and 3% Al_2_O_3_ resulted in poor sinterability. The Al/7% SiC composite’s density, hardness, and microstructure were enhanced by lengthening the sintering time from 30 to 90 min; this change did not affect the Al/SiC/alumina hybrid composites’ compressive strength. Al-based MMCs were fabricated and reinforced with Al_2_O_3_ and ZrO_2_ ceramics utilizing a PM process [30]. The Al-ZrO_2_ composites were harder than the Al-Al_2_O_3_ composites, and hardness of the MMCs rose as wt. % of the ceramic reinforcements increased. The semi-ductile and semi-brittle erosion wear behavior of the composites was observed, with the impact velocity and impingement angle being important factors. Among the composites examined, the 5 wt.% ZrO_2_ reinforcement was determined to be the best. Using PM, a 5-vol% SiC/AA2024 nanocomposite was created, and the ideal manufacturing conditions were found to produce the best tensile characteristics [31]. The microstructure was improved, and SiC nanoparticle dispersion improved by lengthening the ball milling period from 12 to 24 h. This resulted in a notable increase in the 5-vol% SiC/AA2024 nanocomposite’s hardness, yield strength, and ultimate tensile strength. AA7075 metal matrix composites were reinforced with 4, 6, and 10 wt.% SiC particles using PM [32]. As the SiC concentration increased from 0% to 10%, the AA7075-SiC metal matrix composites’ porosity, density, compressive strength, and hardness all improved. Cu-B_4_C MMCs were created via PM. As the B_4_C component rose to 15 wt.%, the Cu-B_4_C composites’ hardness, relative density, and compressive strength also increased [33]. There was good compatibility between the copper matrix and the uniformly dispersed B_4_C particles.

### 2.2. Advances in Liquid-State Processes

This method allows for the fabrication of MMCs by incorporating a metal matrix in liquid form into or with the reinforcement particles. There are various categories into which the utmost liquid phase processing methods can be divided. Infiltration is one of the processes, where a matrix is melted under pressure while the metal is injected in liquid form into the porous preform, retaining the reinforcing phase to produce MMCs. The weight percentages and type of reinforcement have an impact on how vacuum and pressure are applied. Despite the possibility of some porosity and areas with reinforcing aggregate in the completed product, this technique is nonetheless utilized to make Al-based composites. In the reinforcing and matrix interphase, there is little chemical interaction and superior microstructures. The potential for making near-net-shape products and use with a variety of reinforcement materials are some of the key benefits of the infiltration process. Squeeze casting is also known as pressure infiltration or pressure-assisted liquid infiltration of a fibrous or particle material. During the squeeze casting process, liquid metal is squeezed into nearly net-shaped products with the use of an extrusion die and high pressure [34]. Direct and indirect squeeze casting techniques are both used in manufacturing. The melt is supplied into the die using a runner system in the indirect approach. In direct squeeze casting, a tiny shot piston is used to insert the melt into the die, and a runner system is used to apply pressure to the melt.

Stir casting and squeeze casting are popular variants that have been refined with improved mold designs and infiltration techniques to achieve better reinforcement distribution and interfacial bonding. The process of manufacturing particle and fiber-reinforced MMCs is known as stir casting [35]. Since composites are developed through the formation of a vortex, additionally it is called a vortex or slurry casting. For better outcomes, some stir casting techniques employ ultrasonic stirring. To avoid clustering of the fibers or particles, reinforcement materials are appropriately blended and added to the molten metal. The mixture is then either poured into a second die with the proper size or left to cool in the crucible without the mixing arm. It should be noted that adding reinforcing components may increase the viscosity of the molten metals, and this growth in viscosity depends on the proportion of the reinforcement and may make mixing more difficult. Due to the segregation, the finished product could have an uneven microstructure.

Al-based MMCs are primarily produced using solid-phase and liquid-phase manufacturing processes [36]. Stir casting is the most widely used, least expensive, and mass-producible processing method for the manufacture of MMCs. Combining the right components allows MMCs to be built with extraordinary qualities that are hard to obtain with monolithic materials. MMCs’ enhanced qualities, such as their ductility, low weight, and high strength-to-weight ratio, have led to their development for a variety of applications, including defense, aerospace, automotive, and high-temperature situations [37]. Particulate-reinforced composites behave more isotropically, but fiber-reinforced composites offer the greatest increases in strength and stiffness. The mechanical characteristics of Al-MMCs made by the stir casting process are enhanced by the inclusion of reinforcements such as SiC, Al_2_O_3_, TiO_2_, and graphite. These attributes include higher hardness and ultimate tensile strength (UTS). The homogenous spreading of reinforcement particles in a molten metal matrix is determined by stirring duration and speed of the stirrer, which are critical parameters influencing the fabrication of Al-based MMCs (AMCs) via the stir casting method [38]. Pouring temperature, reinforcement size, mold–crucible distance, and pouring pace all have an impact on the casting’s quality and help to keep gas from being trapped. The wettability between reinforcement and matrix is amended by addition of alloying materials and the preheating of the reinforcement and mold. When producing MMCs using the stir casting method, a number of cutting-edge techniques have been used to improve the material’s qualities. Among these techniques is the improvement of mechanical properties like hardness and strength by the adjustment of manufacturing parameters including stirring temperature, duration, and speed. The impact of stirrer blade design on particle distribution is also examined in an effort to improve the reinforcing phases’ dispersion within the matrix. Additionally, through liquid state and semi-solid state mixing processes, the incorporation of ceramic particles into the molten matrix alloy to maximize wettability and yield in MMCs has been investigated, demonstrating advancements in the stir casting technique for the fabrication of components for various industries.

Ceramic reinforcements like Al_2_O_3_ and SiC can be added to MMCs to enhance their mechanical characteristics, such as hardness and grain structure [39]. To obtain a uniform dispersion of the reinforcements and minimize flaws, it is imperative to optimize stir-casting procedure constraints, including temperature, stirring speed, and duration. Fenghong et al. [40] investigated the creation and evaluation of hybrid composites made of an Al-based matrix supplemented with WC and SiC particles to improve the mechanical characteristics of the Al alloy. In contrast to monolithic Al-alloy, SiC, and WC reinforcement particles greatly increased hardness of the Al-based hybrid composites. Because of the stiffer reinforcement particles added, the hybrid composites had greater compressive strength than the monolithic Al alloy. While MMCs manufactured based on Mg and reinforced with several wt.% of fly ash, 2.5, 5, and 7.5%, composite with 7.5 wt.% fly ash had better wear resistance and mechanical properties than the pure Mg [41]. When compared to pure Mg, tensile strength and hardness of Mg-fly ash composites were enhanced by up to 42% and 21%, respectively, with the greatest outcomes observed in samples containing 7.5 wt.% fly ash. Using the stir casting method, AA6061 alloy matrix composites reinforced with 15 and 20 wt.% Si_3_N_4_ were produced satisfactorily [42]. There were no visible indications of porosity or other casting flaws, and the Si_3_N_4_ particles were consistently dispersed throughout the Al alloy matrix. In comparison to parent AA6061 alloy, Si_3_N_4_ reinforcement greatly improved the composites’ microhardness and UTS. A stir casting process was employed to examine impacts of reinforcing Al6061 alloy with 5 wt.% Fe_2_O_3_ and 2–6% B_4_C [43]. Al6061 alloy matrix was supplemented with 5% constant Fe_2_O_3_ and 2–6% variable B_4_C to increase the composite material’s hardness, compressive strength, tensile strength, and microstructural homogeneity. The performance and characteristics of hybrid metal matrix composites (HMMCs) are greatly impacted by rejection phenomena. The rejection of BN-coated SiC particles from aluminum alloys highlights the critical role of ceramic particle stabilization in molten metal, which can impact the homogeneity and efficacy of reinforcement in the composite. The utilization of certain manufacturing processes, specifically liquid-state processing, may give rise to difficulties including particle agglomeration and wettability problems, exacerbating the rejection phenomena and potentially degrading material qualities. Furthermore, during processing, interfacial interactions may cause the matrix to change and reinforcements to deteriorate, which could jeopardize the composite’s mechanical integrity. Notwithstanding these difficulties, HMMCs, especially those reinforced with materials such as SiC and B_4_C, have demonstrated improved mechanical properties under the right processing conditions, suggesting that careful control of rejection events can result in better composites. Thus, maximizing HMMC performance requires a thorough understanding and management of rejection occurrences.

In another study, the impact of adding SiC reinforcement to Al5083 aluminum alloy was examined, and it was concluded that an ideal composition of 7% SiC boosts mechanical, tribological, and microstructure characteristics of the AMCs [44]. In comparison to original Al 5083 alloy, the addition of 7 wt.% SiC increased microhardness by 55%, the ultimate tensile strength by 29%, and the wear resistance and tribological parameters by 25%. It was found that adding SiC reinforcement to the Al matrix gradually increases hardness and UTS from 23 HV to 47 HV and 84 MPa to 130 MPa, respectively, while Al 1100 reinforced with SiC composites varied 0–15 wt.% in 5% increments [45]. Please modify this sentence as ‘Adding Al-TiB_2_ master alloy to molten Mg alloy enhanced hardness, yield strength, UTS, and elongation by 16.1%, 53.0%, 26.3%, and 25.6%, respectively [46]. In the maritime industry and marine conditions, Al 2024 with SiC and ZrSiO_4_ MMCs greatly increases the wear resistance of the as-synthesized matrix alloy; SiC is responsible for the material’s improved surface hardness, and ZiSiO_4_ for its improved bonding strength [47].

## 3. Novel and Modern Methods for MMC Fabrication

**Figure 3:**
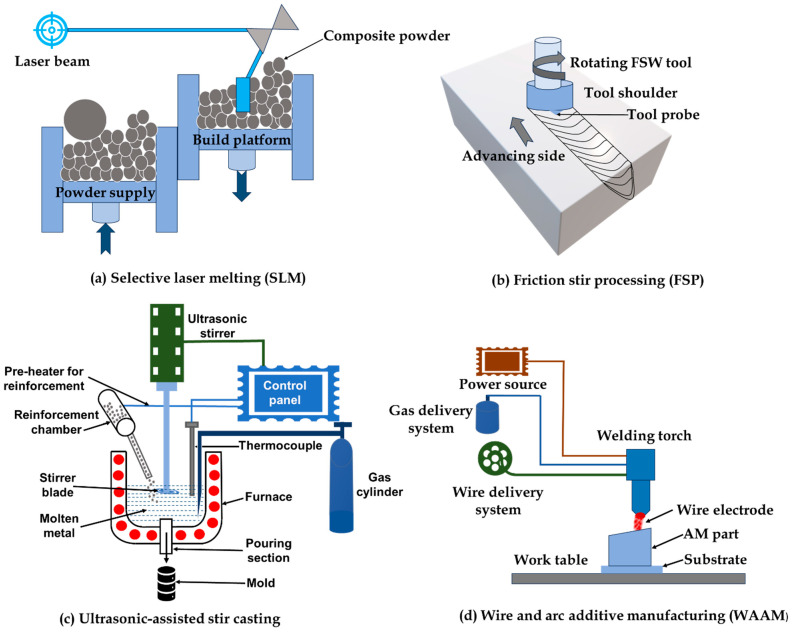
Schematic representation of various novel fabrication techniques for MMCs.

Currently, several cutting-edge processes are being used to fabricate MMCs due to the rising demand for the products made from them. The goal of advanced and novel approaches like selective laser melting (SLM), friction stir processing (FSP), wire arc additive manufacturing (WAAM), and ultrasonic-assisted processing methods is to improve the mechanical potentials and processability of MMCs [48]. For example, a combination of substrate modification in AM and SLM was proposed to create dense, high-strength, and crack-free MMCs for industrial applications. However, FSP methods, such as robotic wire-based FSP, use pre-plasticization procedures and paraxial feeding wires to lower the axial force needed for making complicated parts with good mechanical characteristics and little force. In addition, ultrasonic-assisted processing methods give insights into parameter-dependent mechanics and physical behaviors for better part quality by concentrating on understanding material flow, stress evolution, and microstructure formation during processing of MMCs. Figure 3 illustrates the working principles of various recent novel techniques adopted for the fabrication of MMCs. Ultrasonic-assisted stir casting, FSP, and AM are some of the more advanced methods that make an effort to address the drawbacks of the conventional ways of producing MMCs. Conventional techniques such as stir casting and powder metallurgy have drawbacks, including high melt viscosity, particle agglomeration, and difficulty sintering to a high relative density. Particle agglomeration from stir casting frequently results in uneven reinforcement distribution and early MMC component failure. But FSP and ultrasonic-assisted stir casting increase the homogeneity and uniformity of MMCs by improving the dispersion of reinforcing particles through the use of friction stir methods and ultrasonic waves. Achieving a consistent reinforcement distribution in stir casting is challenging due to high melt viscosity, which can also result in flaws like porosity. By precisely controlling the microstructure, AM techniques lower flaws and raise the overall quality of MMCs. The high cost of PM procedures and their challenges in sintering MMCs to a high relative density raise the total processing expenses. With AM, it is possible to create almost net-shape components straight from raw material, which lowers costs and eliminates the need for intensive secondary processing. Defects like porosity and particle agglomeration are a problem for both stir casting and PM processes, and they can affect the mechanical characteristics of MMCs. By enhancing the dispersion and distribution of reinforcements, FSP and ultrasonic-assisted stir casting methods aid in the reduction of these flaws. The capacity to create intricate forms is constrained; therefore, the design and fabrication of MMC components are highly difficult. The opportunity to create complicated components straight from CAD models is made possible by AM, which enables more creative freedom and the production of intricately shaped components. A summary of recent novel techniques applied for the fabrication of MMCs with the findings is depicted in Table 1.

Laser additive manufacturing (LAM) and other additive manufacturing (AM) processes have various advantages for metallic materials, as well as their potential for advancement in the future [49]. The advantages of AM, particularly LAM, include excellent material usage, quick prototyping, customization, and the capacity to create intricate structures. When it comes to manufacturing precision and the material usage rate, components made by selective laser melting (SLM) outperform those made by laser metal deposition (LMD), while LMD has greater production efficiency. In laser deposition-additive manufacturing (LD-AM), the right choice of process parameters can minimize the negative impacts of excessive thermal stress and component deformation while increasing the melting point and homogenizing the characteristics of the manufactured parts [50]. The LD-AM method shows that it is possible to treat ceramics with high hardness and melting points as well as ceramic-reinforced metal matrix composites. It may also be used to bulk materials as cladding layers to improve surface qualities or add biological or chemical elements. Attar et al. [51] established a framework for employing AM to create porous titanium alloy matrix composites at a low cost for orthopedic implant applications. It describes a process for creating these materials using computer-aided structural design, production optimization, reinforcing techniques, and alloy composition design. Producing titanium composite orthopedic implants with longer lifespans and better structural compatibility is the aim. A novel method, laser powder bed fusion (LPBF), was adopted for fabrication of MMCs of ZrB_2_-fortified Inconel 718 (In718+ZrB_2_) superalloy [52]. Inconel 718+ZrB_2_ outperformed pure Inconel 718 in terms of strength by 10% to 15%, although elongation was somewhat reduced. Furthermore, compared to pure In718, the ZrB_2_ fortified composite showed a notable increase in ductility at 800 °C, achieving about 10% elongation while retaining greater strength. These findings imply that the creation of MMCs doped with ZrB_2_ may greatly improve Inconel 718’s capacity to withstand high temperatures and may even boost the maximum operating temperature limit in systems that now use Inconel 718 components.

Metallic materials with site-specific microstructures may be produced using additive printing, but to fully fulfill this potential, a thorough understanding of microstructure development is required [53]. Solidification structures and post-solidification structures are two categories for the hierarchical microstructures seen in metals produced additively. Supersaturated solid solutions are formed in additive manufacturing due to the high cooling rates, which offer extra strengthening. The primary variables influencing densification behavior of Al-based nanocomposites treated by SLM were the laser power and scan speed [54]. When adopting optimal SLM settings of 350 W laser power and 2.0 m/s scan speed, SLM-processed CNTs/Al-based nanocomposites exhibited exceptional microhardness, tensile strength, and elongation because of their high compaction and ultrafine microstructure. Three strengthening mechanisms were identified as the cause of the superior mechanical properties: load transfer, Orowan looping, and grain refining. A novel technique, paraxial powder injection technology based on wire and arc additive manufacturing (WAAM), was successfully used to build single-layer Ti -based MMCs reinforced with WC particles [55]. The findings showed a gradient distribution of the WC particles over the depth of the layer and that injecting the powders from the back of the molten pool had no effect on droplet transmission. A solid metallurgical connection was established between the matrix and the particles.

High-frequency vibrations are used in ultrasonic-assisted processing to enhance the dispersion of reinforcing particles in the metal matrix. It has been demonstrated that by lowering porosity and strengthening interfacial interaction between matrix and reinforcement, this method improves mechanical characteristics of MMCs. AMCs made by traditional stir casting and ultrasonic-assisted stir casting were compared for their mechanical and physical properties [56]. When SiC particles were added to AMCs, their tensile strength, compressive strength, and hardness rose. These properties were also greater when the ultrasonic-assisted stir-casting technique was used as opposed to the traditional stir-casting method. The electromagnetic stir-squeeze casting technique, when combined with bottom pouring attachments and an ultrasonic probe, is the most promising processing method for MMCs. The benefits of ultrasonic vibrations on the uniform distribution of reinforcements and consequent improvements in mechanical and tribological characteristics in the manufacturing of Mg-based MMCs (MMMCs) using ultrasonic-assisted stir casting have been reviewed in recent research [57]. Although Mg-based alloys offer much potential for use in automotive and aerospace applications, their poor strength, ductility, and wear resistance prevent them from being used widely. The mechanical and tribological characteristics of the MMMCs are enhanced by applying ultrasonic vibrations during the production process to obtain a uniform distribution of the reinforcement particles and decrease the metal matrix’s grain size. A good method for obtaining unvarying spreading of nanoparticles in MMCs is ultrasonic cavitation [58]. Using the ultrasonic cavitation approach, alumina-dispersed aluminum nanocomposites are deprived of discrimination. The utilization of ultrasonic cavitation technology during synthesis of alumina-dispersed aluminum metal matrix composites results in enhanced mechanical and structural characteristics. The development and wear performance of AZ91 Mg matrix composites were improved using Al_2_O_3_ particles produced by stir casting with ultrasonic aid. The development and wear performance of AZ91 Mg matrix composites were improved using Al_2_O_3_ particles produced by stir casting with ultrasonic aid [59]. Al_2_O_3_ particles were uniformly dispersed by ultrasonic agitation, which improved the wear resistance of the Al_2_O_3_/AZ91 composites. The two most important variables influencing the composites’ wear behavior were sliding distance and normal load. The composite with a surface smoothness and wear resistance of 1.5 wt.% Al_2_O_3_ content was the best. The microstructural and mechanical properties were evaluated by incorporating micro and nano Al_2_O_3_ particles into an A356 Al alloy matrix that was manufactured by stir casting and ultrasonic stirring [60]. Strengthening dislocations and reducing grain size, the inclusion of ceramic particle reinforcements, particularly nano-Al_2_O_3_, improves mechanical properties of AMCs. The inclusion of 0.5 wt.% nano-Al_2_O_3_, which had the highest ultimate tensile strength and elongation, resulted in the best mechanical characteristics. Kumar and Kumar [61] investigated effects of T6 heat treatment on properties of base alloy and fabricated composite, as well as microstructural, mechanical, and tribological characteristics of a hybrid AMC that was manufactured utilizing ultrasonic-assisted stir casting with ZrB_2_ and fly ash as reinforcements. The AMC grain refinement was significantly enhanced by the addition of ceramic reinforcements (ZrB_2_ and fly ash). The T6 heat treatment greatly increased the composite’s tensile strength; 5% ZrB_2_ composite demonstrated a tensile strength improvement of 70% over the as-cast composite and a 10% increase over the as-cast base alloy. With a very low COF and strong wear resistance, a composite containing 5% ZrB_2_ demonstrated outstanding tribological characteristics under lubricated circumstances, making it a viable option for use in structural, automotive, and aerospace applications.

An ultrasonic-assisted casting method was utilized to fabricate an Al6061 alloy reinforced with 2 wt.% SiC and different concentrations of Gr (0–3 wt.%) [62]. In contrast to Al6061 alloy, hybrid nanocomposites’ wear rate and COF dropped by as much as 73% and 17.7%, respectively. Using an ultrasonic-assisted stir casting technique, frivolous and wear-resistant AZ91D MMMCs were created and reinforced with TiB_2_ [63]. The most important factor was stirrer speed, followed by ultrasonic power and reinforcement concentration. The optimum combination of low wear and high microhardness was achieved with the following ideal parameters: 1500 W ultrasonic power, 3 wt.% TiB_2_ concentration, and 400 RPM stirrer speed. While fabricating AA6061-B_4_C MMCs using ultrasonic-assisted stir casting, a composite containing 4 wt.% B_4_C had the best mechanical properties, outperforming unreinforced AA6061 matrix by 36.32% in specific UTS, 43.92% in specific compressive strength, 53.41% in specific Vickers hardness, and 50.89% in specific Brinell hardness [64]. Hybrid reinforced composites with varying wt.% of SiC and palm sprout shell ash (PSSA) (0:0, 0:4, 1:3, 2:2, and 4:0 wt.%) were fabricated using the ultrasonic-assisted bottom-poured stir casting technique [65]. The 2:2 wt.% SiC and PSSA hybrid reinforced composites showed a notable improvement in both tensile and flexural strength, with increases in strength of 29.15% and 27.64%, respectively, among all composites.

Friction stir processing (FSP) is a solid-state processing technique that utilizes a non-consumable rotating tool to stir the metal and reinforcement materials, resulting in a fine-grained and homogenized structure. Recent advancements in FSP include the development of multi-pass processing and the integration of ultrasonic vibrations to further refine microstructure and improve mechanical characteristics of MMCs. FSP is an efficient technique for creating AMCs with enhanced mechanical and tribological characteristics [66]. Many processing factors have an impact on the characteristics of the composites made by FSP. Because various Al alloys have varied metallurgical and structural characteristics, their responses to identical FSP processing conditions might vary dramatically. When compared to traditional methods, FSP has become a widely used approach for creating composites and functionally graded systems with better attributes [67]. For sophisticated engineering applications, FSP is a widely used technology to create hybrid MMCs with various reinforcements in addition to mono-reinforced MMCs [68]. The mechanical, hardness, and strength characteristics of MMCs can be amended by adding carbon nanotubes (CNTs) as reinforcements using FSP [69]. The qualities of the finished composite are significantly influenced by the FSP process parameters, which include the number of passes, feed rate, tool rotation speed, and quantity of reinforcement. Additionally, the wear performance, cold formability, and corrosion behavior of the composite may all be enhanced by adding CNTs to the metal matrix. Butola and Murtaza [70] observed how microhardness of AA7075 surface composites was affected by FSP parameters such as tool profile, reinforcements, and tool rotation speed. They found that the most important parameter was tool rotation speed, with 1000 rpm being the ideal speed, and that the best reinforcement for increasing microhardness was B_4_C, which elevated the microhardness of the AA7075 composite by 1.5–1.6 times. With eggshell waste material and SiC/Al_2_O_3_/Ti reinforcement, parent metal AA7075 alloy has favorable properties for FSP [71]. The following process settings are ideal for creating a high-quality FSP composite: tilt angle= 2°, tool speed = 2250 rpm, and reinforcement = Ti + eggshell.

The self-assembled monolayer (SAM) method and FSP were used to add B_4_C nanoparticles, which resulted in a noticeable (more than two-fold) increase in hardness and a minor drop in ductility for the AA6063/B_4_C nanocomposite [72]. Because the AA6063/B_4_C nanocomposite contained hard B_4_C particles, it exhibited better wear resistance than the unreinforced FSPed sample. The B_4_C nanoparticles were distributed uniformly across the treated region thanks to the SAM approach, which also produced consistent mechanical characteristics. FSP was used to effectively create MMCs based on Al, Mg, and copper, using fly ash (FA) as the reinforcement [73]. Because the FSP method effectively stirred the FA particles, they were dispersed evenly throughout the metal matrices with no agglomeration or segregation. All of the composites had higher microhardness once FA particles were added. With unvarying spreading of SiC particles and grain refinement, Al2024 alloy microhardness was enhanced by up to 50% when SiC particles were embedded using FSP methods [74]. The yield strength of the Al2024 alloy as obtained was greatly increased by FSP by a factor of around 2.5, and the yield strength of the MMC sample was further enhanced by the inclusion of SiC particles. In cold-sprayed (CSed) AA2024/Al_2_O_3_ MMCs, FSP causes the Al_2_O_3_ particles to become more refined and dispersed, while faster rotation rates result in more fragmentation [75]. The top surfaces of the friction stir processed (FSPed) MMCs have the maximum level of hardness and refinement, and they display graded microstructure and mechanical characteristics. FSP improves the tensile characteristics (UTS and elongation) of the CSed MMCs, and it can improve them even more at faster rotation rates. The microstructure and mechanical properties of cold-sprayed AA2024/Al_2_O_3_ MMCs were enhanced by FSP; the principal strengthening mechanisms were enhanced interparticle attachment and dispersion consolidation from advanced Al_2_O_3_ elements. Additional FSP passes may help strengthen the composites even more; however, the primary obstacle preventing further improvement of tensile characteristics is still the presence of weak interparticle contacts. Sharma et al. [76] used FSP to examine the wear characteristics of aluminum 7075 alloy reinforced with B_4_C particles. They discovered that the ideal settings are 1600 RPM and 30 mm/min feed rate. Because of the consistent distribution of the B_4_C particles, AMC created by including them had greater wear resistance than both the base material and FSPed alloy without particles. In an innovative way, gas pressure infiltration was successfully used to create a novel interpenetrating MMC with an open porous metallic glass lattice structure, reinforcement phase composed of an alloy composition of Ni_60_Nb_20_Ta_20_, and matrix material, eutectic AlSi12 [77]. The z-direction strength of the Ni_60_Nb_20_Ta_20_-AlSi12 MMC is 430.51 MPa, whereas the x- and y-directions have 280.11 MPa. The MMC reinforced with metallic glass exhibits much-increased strength compared to the pure AlSi12 matrix.

Artificial Intelligence (AI) demonstrates the promise of accelerating the production efficiency of composite materials [78]. Flaw detection in composites, curing composite materials, simulation and process control for composite manufacturing were covered using AI and other digital techniques [79]. These advancements in AI techniques for developing composites with complex and personalized structures can possibly expand the range of applications for composite materials [80]. Machine learning (ML) techniques were used for developing forward models, primarily relating processing parameters to MMC characteristics [81,82]. The composition factors, such as size, and reinforcing particle wt.% as well as processing parameters from different manufacturing processes including casting, powder metallurgy, and mechanical alloying, have been utilized as inputs in ML algorithms to forecast the properties of metal-matrix composites. In addition to predicting qualities, ML techniques may also be used for microstructural analysis and process improvement [83]. Response surface methodology (RSM) and artificial neural network (ANN) techniques were used to optimize the impact of key process factors on the mechanical performance of magnesium metal composites (Mg-MMCs) made by stir casting [84]. The wt.% of particles was the variable with the most influence on the mechanical characteristics of the resulting composites. Based on the desirability function technique, the ideal parameter values for maximum mechanical properties were attained at 312.8 rpm, 11.9 min of stirring duration, and 9.9 wt.% of SiC particle. In a different study, tensile strength and hardness of stir-casted MMCs were mapped against Al6061 alloy and different wt.% of reinforcements using an ANN model [85]. The suggested model’s low mean squared error (0.0058606) and regression value (0.96118) around unity, as demonstrated by the results, indicate its potential to replace conventional experimental methods and cut down on the expense and time required for the investigation of aluminum composites.

Using a stirring-squeeze casting technique, Al alloy was hybridized with Si3N4, Al_2_O_3_, and TiC nanoparticles in a 94% primary material to 6% reinforcing material ratio [86]. The K-nearest neighbor (KNN) and grey wolf optimization (GWO) algorithm-based optimization resulted in a casting die temperature of 400 °C and a squeeze pressure of 100 MPa, yielding tensile strength of 134.75–159.50 MPa and hardness of 80.75–82.92 HBN for the fabricated material. In other important work, ANN-predicted squeeze pressure of 100 MPa, melt temperature of 750 °C, die temperature of 250 °C, and pressure holding duration of 10 s yielded the best results for AA6061/Al_2_O_3_/SiC/Gr composite made by squeeze casting in terms of maximum hardness (131 HVN) and tensile strength (329 MPa) [87]. The combination of ANN-GA technique was applied to optimize the ultrasonic casting conditions for the development of Al-TiB_2_ MMCs [88]. With an error of 2.85%, the developed model demonstrated high accuracy in porosity prediction. The minimal porosity value was 3.88, which corresponds to the ideal casting parameters of a horn depth of 70 mm in the melt, a duration of 20 min for ultrasonication, and a melt temperature of 662.5 °C for ultrasonication. The results indicated that the combined ANN-GA approach may be used to increase the industrial application of particulate Al-based MMCs.

**Table 1:** Summary of some novel and advanced techniques applied for fabrication of MMCs.

Reference	Material and Technique	Findings
[52]	In718/ZrB_2_; 3D printed laser powder bed fusion	Tensile tests at 650 °C exhibited a 10%–15% increase in strength and reduced elongation.
[54]	Al/CNT; Selective laser melting	High microhardness of 154.12 HV and a tensile strength of 420.8 MPa.
[55]	Ti/WC; Wire and arc additive manufacturing (WAAM)	Strong metallurgical bonds between the particles and the matrix were produced with the cost-effective WAAM and paraxial WC powder injection.
[59]	AZ91D/Al_2_O_3_; Ultrasonic-assisted stir casting	29.23% greater wear resistance and 43% higher hardness compared to the AZ91 Mg matrix alloy.
[60]	Al-A356/ micro-Al_2_O_3,_ nano-Al_2_O_3_; Ultrasonic-assisted stir casting	A356 matrix composites containing nano-Al_2_O_3_ showed superior mechanical characteristics compared to micro-Al_2_O_3_ particles.
[61]	AA7075/ZrB_2_/Fly Ash; Ultrasonic-assisted stir casting	The specific wear rate was reduced by 46.94% for the fabricated hybrid Al-based MMCs as compared to the base alloy.
[62]	Al6061/2SiC-xGr; Ultrasonic-assisted stir casting	The wear rate and COF of hybrid nanocomposites decreased by up to 73% and 17.7%, respectively.
[63]	AZ91/TiB_2_; Ultrasonic-assisted stir casting	The ultrasonic agitation of the liquid melt caused uniform dispersion of TiB_2_ reinforcement particles in the composites, which consequently improved the composites’ microhardness and wear.
[64]	AA6061/B_4_C; Ultrasonic-assisted stir casting	The ultrasonication enabled the improvements in specific UTS (36.32%), specific compressive (43.92%), specific VH (53.41%), and specific BHN (50.89%) at such a low 4 wt.% of B_4_C.
[65]	Al-Cu-Mg-alloy/SiC/palm sprout shell ash (PSSA); Ultrasonic-assisted stir casting	With improvements in the strength of 29.15% and 27.64%, respectively, among all composites, 2:2 wt.% SiC and PSSA hybrid reinforced composites demonstrated a significant improvement in both tensile and flexural strength.
[70]	AA7075/B_4_C/SiC/Rice Husk Ash (RHA); Friction stir-processing (FSP)	AA7075/B_4_C composite was found to be 1.5–1.6 times stronger than the base metal; B_4_C was determined to be the most effective reinforcement when compared to SiC and RHA. AA7075/RHA composites exhibited 11% greater microhardness values than the base metal.
[71]	Al 7075/eggshell waste material/ SiC/Al_2_O_3_/Ti; FSP	At the ideal process parameters, RF = Ti + Egg Shell, TS = 2250 rpm, and TA = 20, a range of hardness values was observed for the FSP of the A7075 alloy, using eggshell waste as well as SiC/Al_2_O_3_/Ti reinforcement.
[72]	AA6063/B_4_C; Self-assembled monolayer (SAM) method and FSP	FSPed AA6063/B_4_C nanocomposite showed superior wear resistance due to the presence of hard B_4_C particles. The SAM technique achieved enhanced mechanical properties and homogenous distribution of B_4_C nanoparticles across the treated area.
[73]	AA6061/AZ31, copper/ fly ash; FSP	Regardless of the type of matrix material utilized, FSP is an appropriate technique to generate fly ash-reinforced MMCs.
[74]	Al2024/SiC; FSP	SiC particles embedded in FSPed samples increased microhardness by 50% over the as-received state.
[75]	AA2024/Al_2_O_3_; FSP	UTS and elongation of the cold sprayed (CSed) AA2024/Al_2_O_3_ MMCs were enhanced by FSP, with UTS and elongation increased by up to 27.4% and 25.9%, respectively.
[76]	AA7075/B_4_C and FSP	Ideal parameters of the FSP process, 1600 rotating speed and 30 mm/min feed rate, yielded better wear resistance for the AA7075/B_4_C composites compared to the base material.
[77]	AlSi12/Ni_60_Nb_20_Ta_20_; Gas pressure infiltration process	MMC reinforced with metallic glass exhibits substantially better strength than the pure AlSi12-matrix. The strength of AlSi12/Ni_60_Nb_20_Ta_20_ MMC is 280.11 MPa in the x and y directions and 430.51 MPa in the z direction.
[84]	AZ91/SiC; Stir casting with RSM and ANN approaches	Desirability function analysis showed that 312.8 RPM stirring speed, 11.9 min stirring duration, and 9.9 wt% particle were the ideal parameter conditions for maximal mechanical characteristics. According to ANN and RSM analysis, the wt.% of particles has the greatest influence on the mechanical characteristics of the composites.
[85]	Al 6061/SiC, Gr, Talc, Al_2_O_3_, Fly ash, B_4_C; Stir casted through ANN modelling	With an overall regression (R) value of 0.96118, the model predicted UTS and hardness with a mean squared error of 0.0058606, respectively.
[86]	AA2219/TiC, Al_2_O_3,_ Si_3_N_4_; Stir-squeeze casting with K-nearest neighbor (KNN) and grey wolf optimization (GWO) approaches	Improved tensile strength ranging between 134.75 and 159.50 MPa, and improved hardness ranging between 80.75 and 82.92 HB.
[88]	Al-TiB_2_; Ultrasonic stir casting with ANN, GA approaches	The lowest porosity value of 3.88, which corresponds to the optimal casting parameter, was obtained after applying ANN-GA.

## 4. Conclusions

This review aimed to provide new knowledge related to advancements in the fabrication of MMCs. By examining the state-of-the-art techniques for MMC fabrication, advancements therein, and identifying areas for future research, the paper contributes to the body of knowledge so that future research and development can be pursued by scholars, researchers, and engineers to establish the field further. With the use of several methods such as powder metallurgy and stir casting, the production of MMCs has advanced significantly. These techniques have made it possible to fabricate MMCs with improved mechanical properties, including increased impact resistance, hardness, compressive strength, and tensile strength. Through intensive research, issues related to particle agglomeration, densification, and wettability in MMC production have been resolved, resulting in the development of standardized and sophisticated composite materials. The fabrication of MMCs has seen significant advancements, with traditional techniques being refined and novel methods emerging. The precise control over microstructure and composition offered by these advanced fabrication techniques has the potential to unlock the full potential of MMCs, leading to materials with superior properties for high-performance applications.

The following conclusions can be drawn after analyzing advancements in the fabrication of MMCs:

- Powder metallurgy is gaining popularity as a viable substitute for traditional sand casting and offers several advantages over it, particularly in terms of refining composite potentials. Stir casting is a popular fabrication process for MMCs because of its ease of use, adaptability, and affordability for mass industrial production.
- The aerospace, defense, transportation, and power generating industries have all benefited greatly from advancements in solid-state techniques for manufacturing MMCs. But compared to approaches like powder metallurgy, advances in solid-state techniques like friction stir processing ensure better dispersion of reinforcements, higher densification, and improved interfacial bonding, which eventually produce MMCs with superior physical properties.
- Liquid state processing techniques for MMC production have yielded considerable improvements in terms of ease of use, reduced costs, and enhanced material characteristics. Improvements in MMC fabrication have made it possible to fabricate MMCs with improved tensile, wear, and metallurgical properties in spite of the drawbacks of traditional liquid state methods, such as insufficient particle wetting and the creation of undesired intermetallic particles. The prospective applications of MMCs in sectors such as aerospace, defense, transportation, and power generation have been extended due to these improvements.
- The production of specific parts in industries like aerospace and healthcare has been made possible by advances in additive manufacturing (AM), particularly in metal 3D printing techniques like selective laser melting (SLM). The use of AM techniques for manufacturing MMCs has benefits in terms of cost, time efficiency, precision, and quality.
- In terms of microstructure and mechanical qualities, the ultrasonic-assisted stir casting process has proven to be significantly superior to conventional stir casting processes. It has been concluded that using ultrasonic vibration during the casting process can improve the mechanical properties of MMCs by refining grain size and promoting a more uniform dispersion of reinforcing particles.
- In comparison to conventional processes, the friction stir processing technique fabricates composites that are more promising for a variety of applications due to better strength, hardness, and ductility.
- The fabrication of MMCs is greatly improved by the ANN, GA, and machine learning type intelligent techniques. This leads to improvements in property prediction and process optimization. This technical development is essential to producing high-performing materials with specific applications.
- The development of MMCs using recycled resources, such as borosilicate glass and scrap aluminum, exemplifies a waste-reduction strategy that maximizes mechanical qualities without sacrificing sustainability. This approach encourages ecologically friendly behaviors, which not only enhances material performance but also supports the sustainable development goals.
- Utilizing low-energy sintering techniques in regulated environments can result in low-cost, high-performing composites with decreased porosity. The future of the industry depends on reducing waste and optimizing resource utilization, which in turn supports a circular economy.

The possible future research avenues include accelerating attempts towards employing machine learning for intelligent modeling and optimization, life cycle engineering and analysis of processes, incorporating environmentally benign activities, and utilizing industrial wastes for novel and advanced MMCs fabrication.

## Data Availability

The research data will be made available upon request.

## Nomenclature

Aluminum oxide	Al_2_O_3_
Boron carbide	B_4_C
Carbon nanotubes	CNT
Copper	Cu
Graphite	Gr
Silicon carbide	SiC
Al-based MMC	AMC
Additive manufacturing	AM
Coefficient of friction	COF
Double pressing-double sintering	DPDS
Friction stir processing	FSP
Hot pressing	HP
Silicon nitride	Si_3_N_4_
Titanium diboride	TiB_2_
Titanium oxide	TiO_2_
Tungsten carbide	WC
Zirconium oxide	ZrO_2_
Computer aided drafting	CAD
Mg-based MMC	MMMC
Selective laser melting	SLM
Weight percentage	wt.%
Metal matrix nanocomposites	MMNCs
Ultimate tensile strength	UTS
Friction stir processing	FSP
